# The prestimulus default mode network state predicts cognitive task performance levels on a mental rotation task

**DOI:** 10.1002/brb3.1034

**Published:** 2018-06-22

**Authors:** Tabea Kamp, Bettina Sorger, Caroline Benjamins, Lars Hausfeld, Rainer Goebel

**Affiliations:** ^1^ Department of Cognitive Neuroscience Faculty of Psychology and Neuroscience Maastricht University Maastricht The Netherlands; ^2^ Maastricht Brain Imaging Center (M‐BIC) Maastricht The Netherlands; ^3^ Department of Neuroimaging and Neuromodeling Netherlands Institute for Neuroscience An Institute of the Royal Netherlands Academy of Arts and Sciences (KNAW) Amsterdam The Netherlands

**Keywords:** cognitive task performance, default mode network, mental rotation task, prestimulus activation, prestimulus connectivity

## Abstract

**Background:**

Linking individual task performance to preceding, regional brain activation is an ongoing goal of neuroscientific research. Recently, it could be shown that the activation and connectivity within large‐scale brain networks prior to task onset influence performance levels. More specifically, prestimulus default mode network (DMN) effects have been linked to performance levels in sensory near‐threshold tasks, as well as cognitive tasks. However, it still remains uncertain how the DMN state preceding cognitive tasks affects performance levels when the period between task trials is long and flexible, allowing participants to engage in different cognitive states.

**Methods:**

We here investigated whether the prestimulus activation and within‐network connectivity of the DMN are predictive of the correctness and speed of task performance levels on a cognitive (match‐to‐sample) mental rotation task, employing a sparse event‐related functional magnetic resonance imaging (fMRI) design.

**Results:**

We found that prestimulus activation in the DMN predicted the speed of correct trials, with a higher amplitude preceding correct fast response trials compared to correct slow response trials. Moreover, we found higher connectivity within the DMN before incorrect trials compared to correct trials.

**Conclusion:**

These results indicate that pre‐existing activation and connectivity states within the DMN influence task performance on cognitive tasks, both effecting the correctness and speed of task execution. The findings support existing theories and empirical work on relating mind‐wandering and cognitive task performance to the DMN and expand these by establishing a relationship between the prestimulus DMN state and the speed of cognitive task performance.

## 1 INTRODUCTION

In the past, neuroscientific research has aimed at identifying factors contributing to performance variations on diverse tasks and highlighted the role of prestimulus activation and connectivity in brain regions specific to the task at hand (Colas & Hsieh, 2014; Giesbrecht, Weissman, Woldorff, & Mangun, 2006; Hesselmann, Sadaghiani, Friston, & Kleinschmidt, 2010; Ploner, Lee, Wiech, Bingel, & Tracey, 2010; Sapir, D'Avossa, McAvoy, Shulman, & Corbetta, 2005; Weissman, Roberts, Visscher, & Woldorff, 2006). This evidence for a baseline “preparedness” of task‐related brain regions was soon extended to a more global dimension by uncovering that large‐scale networks constitute different baseline states contributing to different task‐performance levels. More specifically, it has been proposed that spontaneous fluctuations in these networks during the period prior to task onset influence task performance to various degrees, introducing the idea that specific network states affect subsequent performance levels (Colas & Hsieh, 2014; Li, Yan, Bergquist, & Sinha, 2007; Mayhew, Ostwald, Porcaro, & Bagshaw, 2013; Rahnev, Bahdo, de Lange, & Lau, 2012; Sadaghiani, Hesselmann, & Kleinschmidt, 2009; Sadaghiani, Poline, Kleinschmidt, & D'Esposito, 2015; Soravia et al., 2016; Vanhaudenhuyse et al., 2011).

One line of research focused on differentiating an intrinsic and an extrinsic network, both contributing to different levels of awareness (Fox et al., 2005; Fransson, 2005; Tian et al., 2007; Vanhaudenhuyse et al., 2011). While the extrinsic network has been associated with the awareness of external stimuli in the environment, and is fuelled by sensory information, the intrinsic network can be channeled by internally generated mental processes, possibly independent of sensory input (Fox et al., 2005; Lieberman, 2007; Vanhaudenhuyse et al., 2011). Brain regions involved in this intrinsic network have been proposed to comprise regions of the so‐called default‐mode network (DMN) containing hubs in the posterior cingulate cortex (PCC)/precuneus, medial prefrontal cortex (MPFC)/ventral anterior cingulate cortex (vACC) and parietal regions. Cognitive processes driven by these regions involve self‐generated thought, autobiographical memory, mind‐wandering, and daydreaming (Lieberman, 2007; Mason et al., 2007; Scheibner, Bogler, Gleich, Haynes, & Bermpohl, 2017; Smallwood & Schooler, 2015). When these brain regions are active, the mind is therefore involved in internally directed thoughts and external tasks might be difficult to perform when initiated unexpectedly. In fact, it has been proposed that prestimulus activity in the DMN can be linked to subsequent task performance. It was shown that prestimulus DMN activity could predict somatosensory perception (Boly et al., 2007; Mayhew et al., 2013) and auditory stimulus detection (Sadaghiani et al., 2009). More specifically, in studies on prestimulus effects of the DMN on somatosensory and pain perception, Boly et al. (2007) showed that a higher activation in DMN‐related regions before the application of a sensory (thermal) stimulus predicted the conscious perception of the stimulus, compared to no conscious perception. Conversely, there is other evidence for a higher prestimulus activation in the DMN preceding a more intense perception of painful thermal stimulation (Mayhew et al., 2013). In agreement to this study, auditory stimulus detection was shown to be facilitated when the DMN activation was enhanced before stimulus presentation (Sadaghiani et al., 2009).

The DMN has also been linked to cognitive task performance levels, in particular to selective and sustained attention by looking at attentional lapses and response errors. In one early study, it was shown that attentional lapses were associated with a higher task‐induced DMN activation on a selective attention task (Weissman et al., 2006). Furthermore, it has been shown that a higher prestimulus DMN activation precedes errors on a go/no‐go task (Li et al., 2007), as well as errors in a Flanker task (Eichele et al., 2008), compared to correct task performance. Since these early studies, prestimulus DMN effects have been investigated more extensively, showing a more differential role of these effects on task performance. For instance, a higher prestimulus DMN activation was related to a state of attentional stability in a spatial attention task, while a reduced activation was related to a more flexible ability to reallocate attention (Sali, Courtney, & Yantis, 2016). Extending these results, it has been proposed that there are two attentional states which modify cognitive performance levels (Esterman, Noonan, Rosenberg, & DeGutis, 2013), one more stable and less error‐prone state, and one state which leads to suboptimal sustained task performance. Interestingly, the more stable and less error‐prone state was generally characterized by a high DMN activity. At the same time, the chance of errors became more likely when the DMN activation rose beyond a moderate level. Recently, one study has also looked into prestimulus DMN effects on performance in an emotional memory task. In this study, Soravia et al. (2016) showed that a lower DMN activation before the initial encoding of emotional pictures is beneficial for subsequent recognition performance, even 1 week later. In this research, participants were presented with emotional (positive and negative) and neutral pictures and were subsequently tested on the recognition of these pictures. When the DMN activation was comparatively low prior to stimulus presentation, participants were more likely to recognize the pictures in a memory retrieval test 1 week later suggesting that when internally directed mental processes are suppressed, cognitive task processes are facilitated.

The abovementioned studies within the cognitive task domain either used cues in order to indicate an approaching trial (Li et al., 2007; Sali et al., 2016) , or employed event‐related designs with relatively short inter‐stimulus periods (Eichele et al., 2008; Esterman et al., 2013; Soravia et al., 2016; Weissman et al., 2006). Thus, participants were externally cued and thereby triggered into a “task state” (for cued paradigms) or had little time to switch this cognitive state (for paradigms with short inter‐trial times). How the prestimulus DMN state relates to cognitive task performance in a setting which gives participants room to engage in different cognitive states therefore still remains unclear. Therefore, we employed an uncued, sparse event‐related design to investigate potential prestimulus DMN effects on cognitive task performance using the match‐to‐sample mental rotation task, which is a well‐studied classical cognitive task (Shepard & Metzler, 1971). We hypothesized that a lower DMN activation and within‐network functional connectivity would be beneficial for task performance, as in this case, the mind would be more involved in externally‐related thoughts, compared to a higher activation of the DMN during internally‐directed thoughts. We used a match‐to‐sample mental rotation task so that we were able to present short, individual trials. At the same time, trials could be individually adapted, ensuring a constant difficulty level across participants.

Thus, exploring cognitive task performance levels on the grounds of the DMN's state preceding a match‐to‐sample mental rotation task, the current study aimed at investigating.

1. Whether the prestimulus activation and/or connectivity within the DMN can dissociate the correctness of task performance (correct versus incorrect responses), and
2. Whether the prestimulus activation and/or connectivity within the DMN can predict the speed of task execution within the correct responses (fast versus slow trials).

## 2 MATERIALS AND METHODS

### 2.1 Participants

Fourteen healthy volunteers (mean age: 26 ± 2.9 *SD* years, seven female, two left‐handed), with normal or corrected‐to‐normal vision participated in the study. Participants were all students or staff members of the *Faculty of Psychology and Neuroscience* at *Maastricht University*, gave written informed consent before the experimental sessions and were monetarily compensated for their participation. The experimental procedure was approved by the *Ethics Review Committee Psychology and Neuroscience* at *Maastricht University*.

### 2.2 Experimental design

Participants attended two experimental sessions on 2 days, one behavioral pretesting and one fMRI scanning session.

#### 2.2.1 Behavioral pretesting and cognitive task

**Figure 1:**
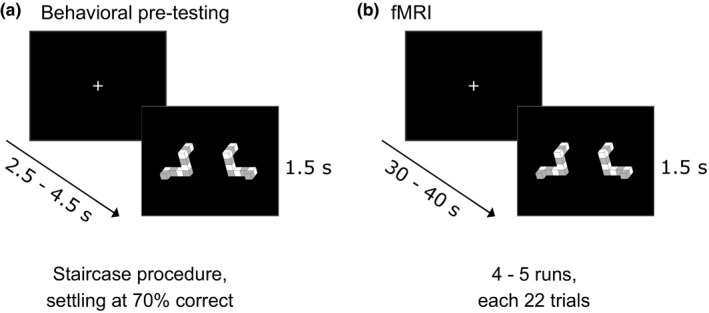
Match‐to‐sample mental rotation task. Participants underwent one behavioral pretesting session, assessing their individual angle rotation at which they scored 70% correct responses on a match‐to‐sample mental rotation task. Trials were presented for 1.5 s, interleaved with a jittered baseline of 2.5 to 4.5 s (a). In the subsequent fMRI session, four to five runs of the match‐to‐sample mental rotation task were performed in a sparse event‐related design. 22 trials were presented, interleaved with long baseline periods, jittered between 30 and 40 s (b)

During the 20 min pretesting, volunteers performed a match‐to‐sample mental rotation task (Shepard & Metzler, 1971). This pretesting was used to ensure that participants were able to perform the task, as well as identifying the individual difficulty level (angle rotation) for keeping performance across participants identical (aiming at 70% correct trials). Participants were visually presented with two images of three‐dimensional (3D) objects on a black background (Figure 1) taken from a mental rotation stimulus library (Peters & Battista, 2008). In each trial, the two objects were either rotated, but identical shapes (same object), or rotated and mirrored shapes (different object) of one another and were presented for 1.5 s, followed by a baseline period varying per trial between 2.5 and 4.5 s (Figure 1). Participants were asked to indicate whether the two objects were the same (just rotated) or different (additionally mirrored) shapes by button presses as fast and as accurately as possible. As participants previously showed a variation in cognitive ability on mental rotation tasks and the difficulty level can be manipulated based on increasing the angle rotation between the two objects (Peters & Battista, 2008; Shepard & Metzler, 1971), the angle rotation at which each individual participant scored 71% correct on average was identified based on a staircase procedure. The staircase procedure was a simple two‐up, one‐down procedure, increasing the angle rotation difference between the two objects by 10° each time the participants responded correctly two times in a row, and lowering the angle rotation difference by 10° when one incorrect response was made.

#### 2.2.2 fMRI session

After informing participants about the experimental procedure, anatomical images were acquired, followed by a 15 min resting state run in order to identify individual DMNs. During the resting state run, participants were asked to keep their eyes open, fixating a fixation cross and to not think of anything in particular. Four to five runs of the match‐to‐sample mental rotation task were administered consecutively to investigate prestimulus effects of the DMN state on performance levels. Different from the pretesting session, trials were presented in a sparse event‐related design, with inter‐stimulus intervals ranging between 30 and 40 s, so that each run consisted of 22 trials in total (resulting in a run length of around 14 min 30 s). These long baseline periods were chosen so that participants could freely engage in different cognitive states. Moreover, they ensured that the blood oxygenation level dependent (BOLD) response went back to baseline and eliminated any carry‐over effects from the previous trials. During the baseline periods, participants were asked to focus on a fixation cross and to not think of anything in particular. The first trial of each participant used the angle rotation between the two objects at which they scored 70% correct at the end of the staircase procedure of the pretesting session. This was done to ensure that participants were able to do the task, but at the same time ensuring that there were a sufficient amount of incorrect trials for subsequent analyses.

### 2.3 Data acquisition

Anatomical and functional MRI data were acquired using a 3T whole‐body MAGNETOM PrismaFit scanner (Siemens AG, Erlangen, Germany). Participants were comfortably placed in the scanner and their heads were comfortably padded with foam to avoid head motion. T1‐weighted anatomical scans were obtained for each participant using a three‐dimensional magnetization‐prepared rapid‐acquisition gradient‐echo (MPRAGE) sequence (192 slices, slice thickness = 1 mm, no gap, TR = 2,250 ms, TE = 2.21 ms, FA = 9°, FOV = 256 × 256 mm^2^, matrix size = 256 × 256, total scan time = 8 min 26 s). Functional images were acquired using multiband (MB) accelerated echo‐planar imaging (EPI) (Moeller et al., 2010), obtaining one resting state run (900 volumes, 48 slices, voxel dimensions = 2.5 mm isotropic, no gap, TR = 1,000 ms, TE = 31 ms, FA = 62°, FOV = 224 × 224 mm^2^, matrix size = 89.6 × 89.6, MB factor = 4, slice order = interleaved) and four to five mental rotation task runs (equal scanning parameters, 880 volumes).

### 2.4 Data analysis

#### 2.4.1 Behavioral data

The responses of each participant were sorted *post hoc* in two different ways:

1. Correctness of task performance: In order to investigate whether correct trials could be distinguished from incorrect trials based on the prestimulus DMN state, trials were sorted accordingly. The percentage of correct responses for each individual participant was calculated and assessed in terms of suitability for further analysis. As a result, participants were excluded from this analysis when scoring at around chance level (below 60% correct on average) or when having too little trials with an incorrect response (scoring above 90% correct).
2. Speed of correct task performance*:* To determine whether the prestimulus DMN state could dissociate different levels of correct task performance, correct response trials were sorted into fast and slow trials. As the angle rotations varied across participants (due to the staircase procedure and individual performance on the task), a median split of the reaction times was performed for each angle rotation per participant.

Trials with a response time longer than 1.5 s after stimulus offset, as well as trials with missing responses were excluded from further analyses.

#### 2.4.2 Preprocessing of imaging data

All preprocessing and analyses of the imaging data were performed with BrainVoyager 20.4 (BrainInnovation, Maastricht, the Netherlands). Anatomical images were corrected for spatial intensity inhomogeneity and subsequently normalized into MNI (Montreal Neurological Institute) stereotactic space. Functional data were preprocessed using 3D motion correction, slice scan time correction, linear trend removal and a temporal high‐pass filter (cut‐off value: 0.008 Hz).

#### 2.4.3 Individual DMN definition

The individual DMNs were identified based on the data from the resting state run using the following steps:

1. Two individual seed‐based analyses were performed for each participant using seeds of 10 mm spheres in two main nodes of the DMN, the posterior cingulate cortex (PCC)/precuneus (*x* = −5, *y* = −49, *z* = 40) and the medial prefrontal cortex (MPFC)/ventral anterior cingulate cortex (vACC)( *x* = −1, *y* = −47, *z* = 4). The respective coordinates were selected based on a meta‐analyses about these hubs analysed from resting state data (Fox et al., 2005). For these seed‐based analyses, a general linear model (GLM) was calculated, including the time course of the PCC/precuneus or MPFC/vACC as the main predictor, as well as the realignment parameters (three rotations and three translations), the signal from the white matter, and the signal from the ventricles as confounding predictors. The connectivity maps were consecutively corrected for multiple comparisons using cluster‐size thresholding (with an initial threshold of *p* < 0.001) and the individual DMN was defined as the overlap of the resulting two maps.
2. A group DMN mask was created by applying the abovementioned steps on the group level, performing a random effects GLM analysis across data from all participants. The individual DMNs were subsequently masked with the group result.
3. In order to concentrate on the areas of the DMN which showed a negative task‐induced activation and exclude extrinsically related areas (e.g., visual cortex), we further constrained the networks with the task‐negative network of each participant using the mental rotation task runs (GLM analysis, contrast mental rotation versus baseline, *p* < 0.001, uncorrected).

#### 2.4.4 Prestimulus DMN activation

In order to assess differences in prestimulus activation patterns within the whole DMN across task performance levels, a random effects group GLM was performed within the individual DMNs. The GLM included predictors for correct and incorrect responses (or fast and slow trials) and four stick predictors covering the peri‐stimulus period from −2 to +1 s. A paired‐samples *t*‐test was carried out for correct versus incorrect responses (fast trials versus slow trials) at time point 0 (stimulus onset). To further investigate whether either of the two DMN hub regions drives potential prestimulus effects, we carried out the same analysis based on the PCC/precuneus and the MPFC/vACC alone (with individual hubs defined as under section 2.4.5).

Additionally, a linear contrast analysis was performed across the four stick predictors within the whole DMN in order to test the propagation of the DMN effects over time while approaching a correct versus an incorrect trial (fast versus slow trial). A linear contrast analysis tests for a hypothesized linear pattern in the data. For this, the incorrect (slow) beta values for each time point entering the analysis (*t* = −2, −1, 0, +1) were subtracted from the corresponding correct (fast) beta value. A linear contrast was defined by assigning contrast coefficients (−3, −1, +1, +3) to the resulting difference values. This contrast was subsequently tested against 0 to investigate a linear pattern (α = 0.05).

This peri‐stimulus time window was chosen for the linear contrast analysis as previous research used time points just prior, at stimulus onset or one time point after stimulus onset (as the hemodynamic delay does not cause an effect of stimulus onset on the BOLD response at this time point, compare Hesselmann et al., 2010) to investigate prestimulus effects (e.g., Coste, Sadaghiani, Friston, & Kleinschmidt, 2011; Esterman et al., 2013; Giesbrecht et al., 2006; Hesselmann et al., 2010; Mayhew et al., 2013; Sadaghiani et al., 2009; Soravia et al., 2016). Hereby, the effects can be reliably disentangled from the previous trial and can be interpreted in light of the momentary DMN state just before task onset.

#### 2.4.5 Prestimulus within‐DMN connectivity

A beta series correlation analysis (Rissman, Gazzaley, & D'Esposito, 2004) was implemented to investigate the differences in task performance with regard to the functional connectivity prior task onset between the two main hubs of the DMN (PCC/precuneus and MPFC/vACC) in a region‐of‐interest approach. The analysis was done by applying the following steps:

1. The PCC/precuneus and the MPFC/vACC were identified in each hemisphere as 4 mm spheres surrounding the lowest activation point during the mental rotation task for each participant, masked with the individually defined DMN (resulting in four regions of interest). As a prerequisite, the chosen voxels for these two main hubs needed to be correlated significantly.
2. A GLM was calculated including predictors for the task performance conditions (correct versus incorrect responses or fast versus slow trials) and a finite impulse response (FIR) model with stick predictors for the prestimulus period −2 to 0 s.
3. Single‐trial beta values on the basis of the GLM analysis were extracted for the prestimulus period for all four regions and averaged across hemispheres for PCC/precuneus and MPFC/vACC respectively.
4. The resulting single‐trial betas between PCC/precuneus and MPFC/vACC hubs were correlated for correct and incorrect responses (fast trials and slow trials) separately.
5. A paired‐samples Wilcoxon signed‐rank test was performed on the Fisher *z*‐transformed correlation coefficients in order to test the difference between correct versus incorrect responses (fast trials versus slow trials) (α = 0.05).

## 3 RESULTS

### 3.1 Behavioral results

Participants performed the mental rotation task during the MRI session with an average percentage of correct responses of 76.5% (±11.2 STD). One participant was excluded from further analyses because he did not exceed chance level performance (47.73% correct responses). Thus, data of 13 participants were used for the fast versus slow trial analyses. As two participants had too few incorrect responses in order to compare correct versus incorrect trials (4.1% and 9.1%), data of 11 participants were included for the correct versus incorrect response analyses.

### 3.2 Individual DMN definition

**Figure 2:**
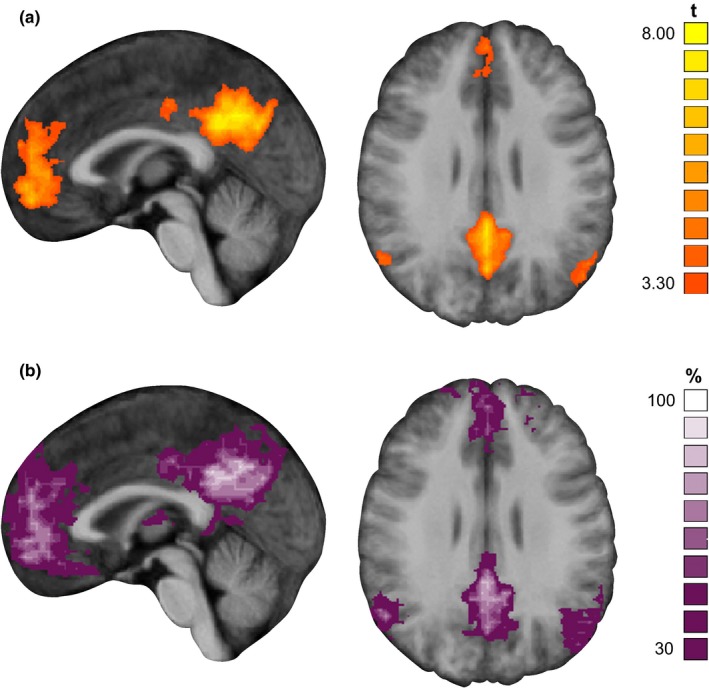
Result of DMN definition. (a) Group average of the DMN obtained from the resting state random effects analysis of all participants included in the fast versus slow trial analysis (cluster‐size thresholded at *p* < 0.001) overlaid on slices of the participants' mean anatomy in MNI space. (b) Probabilistic map of individual DMN definitions showing the percentage of spatial overlap of the individual DMN definitions across participants. Remarks. Left panels: sagittal view, right panel: transversal view, MNI coordinates: *x* = 0, *z* = 28

For each participant, an individual DMN could be identified based on the resting state run and the objective procedure described above. Figure 2 shows the group average of the individual networks (Figure 2a) and the probabilistic map of all individual DMNs (Figure 2b).

### 3.3 Prestimulus DMN activation

**Figure 3:**
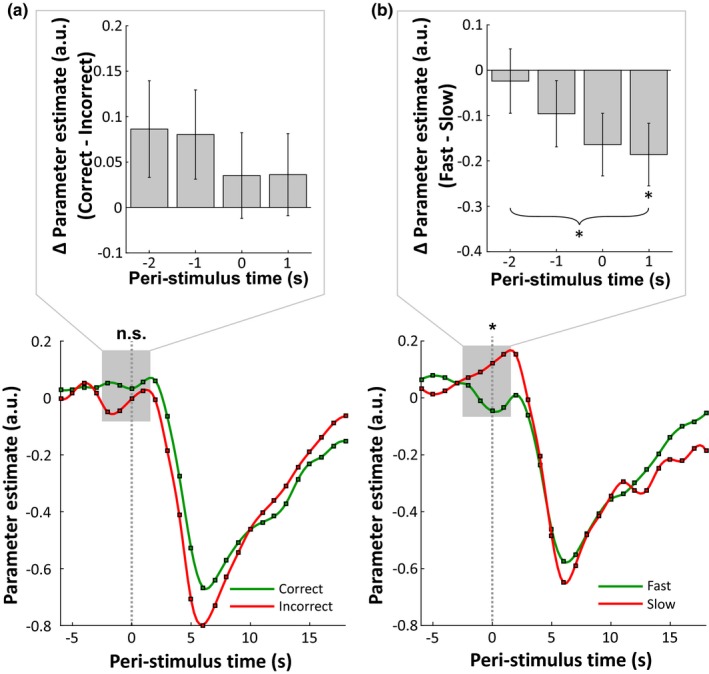
Prestimulus DMN activation for both correctness and speed of task performance analyses. (a) Upper panel: The difference of obtained peri‐stimulus beta values and standard error, comparing correct versus incorrect responses in the DMN within the tested time window (peri‐stimulus time points −2 s until +1 s). Prestimulus activation at time point 0 was not significant (*t*[10]  = 0.63, *p* = 0.27, paired‐samples *t*‐test, one‐sided). Also the linear contrast analysis across the tested time window did not reach significance (*F*[1,10]  = 0.89, *p* = 0.368). Lower panel: Peri‐stimulus beta time series averaged across all individual DMNs surrounding stimulus onset (indicated by grey dashed line) for correct (green curve) and incorrect (red curve) trials. The grey rectangle depicts the time window used for testing. The *x*‐axis represents the peri‐stimulus time period (in s), while the *y*‐axis depicts the parameter estimates in arbitrary units (beta values). (b**)** Upper panel: The difference of obtained peri‐stimulus beta values and standard error, comparing fast versus slow trials in the DMN within the tested time window (peri‐stimulus time points −2 s until +1 s). Prestimulus activation at time point 0 was significant (*t*[12]  = −2.36, *df* = 12, *p* = 0.018, paired‐samples *t*‐test, one‐sided) with a higher activation in the DMN prior slow trials, indicated by the asterisk. Furthermore, the linear contrast analysis showed a significant linear decrease in the difference of fast‐slow trials (*F*[1,12]  = 6.22, *p* = 0.028, depicted by curly bracket).Lower panel: Peri‐stimulus beta time series averaged across all individual DMNs surrounding stimulus onset (indicated by grey dashed line) for fast (green curve) and slow (red curve) trials. The grey rectangle depicts the time window used for testing. Remark. *Δ = difference*

The prestimulus DMN activation did not dissociate between correct and incorrect responses as there was no difference in the activation of the DMN at stimulus onset (Figure 3a, *t*[10]  = 0.63, *p* = 0.27, paired‐samples *t*‐test, one‐sided). Moreover, neither the prestimulus PCC/precuneus, nor the MPFC/vACC activation could differentiate correct from incorrect responses (PCC/precuneus: *t*[10]  = −0.231, *p* = 0.41, paired‐samples *t*‐test, one‐sided; MPFC/vACC: *t*[10]  = −0.333, *p* = 0.37). Additionally, the related linear contrast analysis did not yield significance (*F*[1,10]  = 0.89, *p* = 0.368). However, the activation in the individual DMNs prior to stimulus presentation was predictive of correct task performance levels, varying between fast and slow trials. Before fast trials, the DMN showed a reduced activation in comparison to slow trials (Figure 3b, *t*[12]  = −2.36, *df* = 12, *p* = 0.018, paired‐samples *t*‐test, one‐sided). Furthermore, both hub regions of the PCC/precuneus and MPFC/vACC separately showed a significantly lower prestimulus activation for fast compared to slow trials (PCC/precuneus: *t*[12]  = −1.947, *p* = 0.038, paired‐samples *t*‐test, one‐sided; MPFC/vACC: *t*[12]  = −1.856, *p* = 0.044, paired‐samples *t*‐test, one‐sided). Moreover, linear contrast analysis showed that for the fast versus slow analysis, there was a significantly increasing difference in the activation of the DMN between fast and slow trials the closer the time point was to stimulus presentation (*F*[1,12]  = 6.22, *p* = 0.028).

### 3.4 Prestimulus within‐DMN connectivity

**Figure 4:**
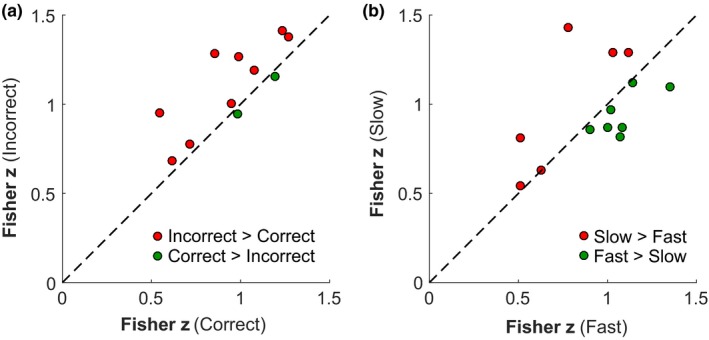
Prestimulus within‐DMN connectivity for both correctness and speed of task performance analyses. Results of the beta series correlation of main hubs of the DMN for the correctness of task performance (a) and speed of task performance analyses (b). Each dot represents the two Fisher *z*‐transformed correlation coefficients between PCC/precuneus and MPFC/vACC for the prestimulus period of the respective analysis for an individual participant, i.e., dots falling above the dashed line represent participants with a higher DMN‐connectivity for incorrect (slow) trials (red dots), whereas dots below the dashed line represent participants who showed a higher within‐DMN connectivity for correct (fast) trials (green dots). The Wilcoxon signed rank test of correct versus incorrect responses was significant (*p* < 0.008), with a higher DMN‐connectivity prior to incorrect versus correct trials

Differences of within‐DMN connectivity states predicted correct and incorrect responses with a lower connectivity between PCC/precuneus and MPFC/vACC preceding correct trials than incorrect trials (Figure 4a, Wilcoxon signed rank test, *Z* = −2.67, *p* < 0.008). However, the same did not hold for the fast versus slow trial analysis, as no connectivity differences between the two trial types could be observed within the DMN prior task onset (Figure 4b, Wilcoxon signed rank test, *Z* = −0.094, *p* = 0.925).

## 4 DISCUSSION

The aim of the present study was to investigate whether the DMN state prior to task onset is predictive of cognitive task performance levels on a mental rotation task (note that the word predictive is here and in the following meant in a correlational, not causal sense). To do this, we employed a sparse event‐related fMRI design, evaluating activation and connectivity levels within the DMN prior to the onset of a match‐to‐sample mental rotation task with regard to task performance levels. The study revealed two major relationships between the prestimulus DMN state and task performance. First, the amplitude of prestimulus DMN activation is associated with correct task performance levels, being able to predict the speed of task performance. The whole‐network activation analysis showed that before fast, correct trials, the DMN activation was lower than before slow correct trials (Figure 3). Furthermore, the difference between the fast and slow prestimulus DMN activation became more pronounced until trial onset (Figure 3b, linear contrast analysis). Second, the within‐DMN connectivity preceding trial onset could predict the correctness of task performance. The connectivity analysis showed that before correct task execution, the two major DMN hubs PCC/precuneus and MPFC/vACC were correlated less than before incorrect task performance (Figure 4a). Taken together, these complementary results for prestimulus activation and connectivity effects on cognitive task performance suggest that both types of analysis are sensitive for different task effects. Therefore, future studies should look on both types of analysis in order to better understand the DMN's role in cognition.

The results of this study are in line with empirical work, as well as theoretical considerations on the DMN network function and cognitive task performance. Previous research investigating the role of the DMN preceding task performance supports our findings that a lower prestimulus DMN state is beneficial for cognitive task performance (Eichele et al., 2008; Esterman et al., 2013; Li et al., 2007; Sali et al., 2016; Soravia et al., 2016; Weissman et al., 2006). In the current study, participants performed the mental rotation task in a sparse event‐related design and were thus not presented with visual input for a longer period. Thereby, they were flexible to engage in different cognitive modes. A similar sparse event‐related design as employed in the current study measured DMN activation before a Stroop task (Coste et al., 2011). While the authors did not find prestimulus DMN effects, results indicated a trend for a decreased activation in DMN‐related regions for faster trials on this cognitive task compared to slower trials. Thus, our findings extend prior research by showing that in a cognitive task with a low frequency of task trials, cognitive performance is enhanced when participants are engaged in a state of relatively low DMN activation just prior to a task trial. Furthermore, we show that not only a decreased activation level, but also a decreased connectivity within the DMN can lead to better task performance on a cognitive task. This supports the idea that fluctuations in the DMN might be meaningful for subsequent task performance, establishing dynamic states and herewith contributing to differential task outcomes. However, previous studies suggest that the nature of the relationship between the DMN and task performance has not been uniquely established. While the abovementioned studies show converging evidence for a beneficial role of a reduced DMN state before cognitive task onset, a number of other studies have recently shown a more variable role of the DMN for sensory tasks (Boly et al., 2007; Mayhew et al., 2013; Sadaghiani et al., 2009). These studies employed near‐threshold sensory tasks, on the one hand showing a lower prestimulus activation or connectivity predicted enhanced levels in detection performance (Boly et al., 2007), on the other hand showing a higher prestimulus DMN activation for more intense perception (e.g., more sensitive auditory stimulus detection, more extreme perception during thermal stimulation) of sensory stimuli (Mayhew et al., 2013; Sadaghiani et al., 2009, 2015). However, the tasks at hand relied primarily on processing in sensory areas, rather than higher‐order cognitive areas. The DMN might therefore play different roles with regard to task performance when integrating sensory and higher‐order cognitive processes. In contrast to sensory tasks, cognitive tasks such as the match‐to‐sample mental rotation task employed in the current study might increase the need to distribute connections across different modules instead of relying on network integrity (Sadaghiani et al., 2015). It might be possible that within diverse DMN states, the brain benefits from a less integrated DMN (manifested in less functional connectivity within the network) in order to respond optimally to a changing environment. It is therefore conceivable that the difference found between the abovementioned studies and the current study might be caused by the task at hand (sensory versus cognitive tasks). In order to reconcile the variable findings within the sensory task domain and to relate prestimulus DMN effects in both sensory and cognitive tasks, a future study could employ stimuli which permit both a cognitive and a sensory task for the participant. Such a study would have the power to investigate the DMN's role in both sensory and cognitive task performance, while keeping sensory input constant.

The decreased DMN activation and connectivity between PCC/precuneus and MPFC/vACC preceding task onset can be interpreted in light of the literature on mind‐wandering and its relationship to the DMN. In recent years, it has been shown that levels of DMN activation are linked to the frequency and depth of mind‐wandering (Christoff, Gordon, Smallwood, Smith, & Schooler, 2009; Mason et al., 2007; Scheibner et al., 2017; Smallwood & Schooler, 2015). Hereby, mind‐wandering was defined as any kind of cognition independent of the task at hand, involving processes like retrieval of autobiographic memory, future planning or evaluating and judging the present (Scheibner et al., 2017). Mind‐wandering has been associated with several impairments in cognitive functioning (for a review, see Smallwood & Schooler, 2015), however, a reduction in mind‐wandering, as well as concurrent decrease in the DMN could be observed when applying meditation strategies (Berkovich‐Ohana, Harel, Hahamy, Arieli, & Malach, 2016; Scheibner et al., 2017). Furthermore, these behavioral and neural changes in the DMN were associated with an increase in performance on working memory tasks (Berkovich‐Ohana et al., 2016). The current project supports this idea by showing that potential mind‐wandering episodes (as represented by a heightened DMN activation/connectivity in the baseline period just before task onset) might lead to decreased task performance. Furthermore, spontaneous fluctuations of task modes have been shown to occur at around 20 s, and fluctuations of external versus internal awareness were correlated with DMN activation variability (Vanhaudenhuyse et al., 2011). In the current study, introducing long interstimulus intervals of 30–40 s with no task‐related stimulation in an otherwise stimulus‐deprived environment of the MRI scanner made it possible for participants to switch task modes, potentially shifting from task states to mind‐wandering phases. This suggests that there may be a direct link between mind‐wandering, activation in the DMN and behavioral performance on cognitive tasks, opening up potential scenarios for training cognition and performance levels by, for example, meditation practices. However, it is not clear whether participants were mind‐wandering more actively in the slow or incorrect trials compared to the fast or correct trials. Therefore, introducing thought probes after the individual trials in order to measure mind‐wandering or task‐related thoughts should be introduced in future studies in order to make direct inferences about mind‐wandering preceding task onset and poor task performance.

Another consideration with regard to the current study concerns the analysis of incorrect trials. Prestimulus activation in the DMN could dissociate fast from slow correct trials, however, did not predict whether a trial was performed correctly or incorrectly (Figure 3a). While in correct trials, performance can be evaluated on the basis of the cognitive processes taking place (by looking at differential task performance in the realm of reaction times), this is not possible with regard to the incorrect trials, where we do not have the possibility to draw conclusions about the cognitive processes at hand. Moreover, the current study looked at prestimulus DMN effects on correctness of task performance as well as speed of correct task execution, which is why the study design used a staircase procedure to keep the difficulty level at around 70% correct responses, ensuring that also incorrect trials could occur. As a result of this, two restrictions were induced: the amount of potential correct task responses was limited and the angle difference between the two objects varied across trials for each participant (making the reaction times dependent on the angle difference in each trial, as there is a positive association between angle difference and reaction times (Shepard & Metzler, 1971)). In order to increase the power and potentially fine‐grain the analysis on different clusters of reaction times, future studies should employ a cognitive task which makes reaction times comparable across trials or should focus the design on correct trials and keep the angle difference between the objects constant. By doing this, reaction times could be used as a regressor in the analysis instead of transforming them into categories of fast and slow trials. In this case, it would be possible to investigate whether the prestimulus DMN state has an even more elaborate prediction strength of differential task outcome, by being able to differentiate across a continuum of responses.

In conclusion, the current study emphasizes the role of the prestimulus DMN state in task performance, by showing that the activation and within‐network connectivity of the DMN can predict the response speed and the correctness respectively. These results provide further evidence for large‐scale network influences on cognitive behavior and underlines the importance of investigating prestimulus activation and connectivity effects within the DMN in a variety of tasks.
