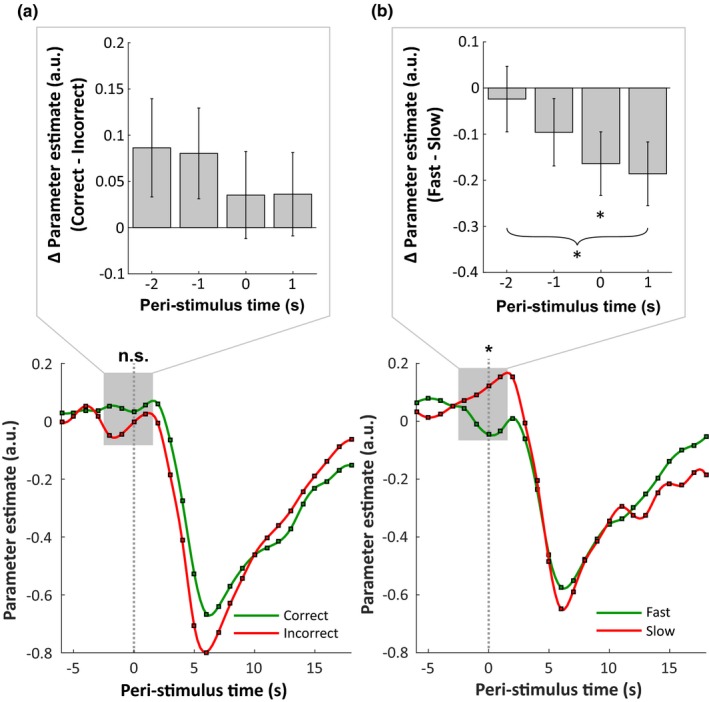# The prestimulus default mode network state predicts cognitive task performance levels on a mental rotation task

**DOI:** 10.1002/brb3.1186

**Published:** 2018-12-26

**Authors:** Tabea Kamp, Bettina Sorger, Caroline Benjamins, Lars Hausfeld, Rainer Goebel

In Kamp, Sorger, Benjamins, Hausfeld, and Goebel (2018), the following errors have been published:

- In Abstract, the word “higher” in the first sentence of the Result section should be changed to “lower,” so it reads “We found that prestimulus activation in the DMN predicted the speed of correct trials, with a lower amplitude preceding correct fast response trials compared to correct slow response trials.”
- In Figure 3b, the asterisk under time point 1 should be placed under time point 0. Below is the updated figure.